# A practical method to quantify knowledge‐based DVH prediction accuracy and uncertainty with reference cohorts

**DOI:** 10.1002/acm2.13199

**Published:** 2021-02-26

**Authors:** Brent M. Covele, Cody J. Carroll, Kevin L. Moore

**Affiliations:** ^1^ Radiation Medicine and Applied Sciences University of California – San Diego La Jolla CA USA; ^2^ Department of Statistics University of California – Davis Davis CA USA; ^3^ Radiation Medicine and Applied Sciences University of California – San Diego La Jolla CA USA

**Keywords:** DVH error, DVH estimate, knowledge‐based planning, ORBIT‐RT

## Abstract

The adoption of knowledge‐based dose‐volume histogram (DVH) prediction models for assessing organ‐at‐risk (OAR) sparing in radiotherapy necessitates quantification of prediction accuracy and uncertainty. Moreover, DVH prediction error bands should be readily interpretable as confidence intervals in which to find a percentage of clinically acceptable DVHs. In the event such DVH error bands are not available, we present an independent error quantification methodology using a local reference cohort of high‐quality treatment plans, and apply it to two DVH prediction models, ORBIT‐RT and RapidPlan, trained on the same set of 90 volumetric modulated arc therapy (VMAT) plans. Organ‐at‐risk DVH predictions from each model were then generated for a separate set of 45 prostate VMAT plans. Dose‐volume histogram predictions were then compared to their analogous clinical DVHs to define prediction errors $V_{clin,i}‐V_{pred,i}$ (*i*th plan), from which prediction bias *μ*, prediction error variation *σ*, and root‐mean‐square error $RMSE_{\mathrm{pred}}\equiv \sqrt{\frac{1}{N}\sum_{i}{\left(V_{clin,i}‐V_{pred,i}\right)}^{2}}≅\sqrt{\sigma^{2}+\mu^{2}}$ could be calculated for the cohort. The empirical $RMSE_{\mathrm{pred}}$ was then contrasted to the model‐provided DVH error estimates. For all prostate OARs, above 50% Rx dose, ORBIT‐RT *μ* and *σ* were comparable to or less than those of RapidPlan. Above 80% Rx dose, *μ* < 1% and *σ* < 3‐4% for both models. As a result, above 50% Rx dose, ORBIT‐RT $RMSE_{\mathrm{pred}}$ was below that of RapidPlan, indicating slightly improved accuracy in this cohort. Because *μ* ≈ 0, $RMSE_{\mathrm{pred}}$ is readily interpretable as a canonical standard deviation *σ*, whose error band is expected to correctly predict 68% of normally distributed clinical DVHs. By contrast, RapidPlan's provided error band, although described in literature as a standard deviation range, was slightly less predictive than $RMSE_{\mathrm{pred}}$ (55–70% success), while the provided ORBIT‐RT error band was confirmed to resemble an interquartile range (40–65% success) as described. Clinicians can apply this methodology using their own institutions’ reference cohorts to (a) independently assess a knowledge‐based model's predictive accuracy of local treatment plans, and (b) interpret from any error band whether further OAR dose sparing is likely attainable.

**Abbreviations**
KBP: knowledge‐based planning
OAR: organ‐at‐risk
DVH: dose‐volume histogram
Rx: prescription
ORBIT‐RT: Online Real‐Time Benchmarking Information Technology for RadioTherapy
VMAT: volumetric modulated arc therapy
RMSE: root‐mean‐square error
IQR: interquartile range

## 1 INTRODUCTION

Knowledge‐based dose estimation models have demonstrated utility in patient‐specific treatment plan quality control and in knowledge‐based planning (KBP) systems, whereby the treatment planning process is automated by setting optimization objectives for organ‐at‐risk (OAR) sparing based on patient‐specific dose predictions. Models are trained on past treatment plans to predict what is likely achievable for a new patient's OAR dose‐volume histograms (DVHs). There have been several knowledge‐based OAR DVH estimation methods described in the literature,^1^, ^2^, ^3^, ^4^, ^5^, ^6^, ^7^, ^8^, ^9^, ^10^ all utilizing quantification of anatomic features and correlation to resultant plan dosimetry, but the algorithmic details vary from model to model.

The proliferation of different knowledge‐based models underscores the need to set expectations for DVH prediction accuracy in a transparent way. From the perspective of a user, ideal knowledge‐based DVH predictions should (a) exhibit minimal systematic bias relative to the clinically accepted DVHs, and (b) quantify their uncertainty in terms of familiar, readily interpretable metrics, such as standard deviation. However, it is often unclear how to interpret DVH prediction error as a confidence interval for a percentage of clinically acceptable DVHs, if such an error band is provided at all. Clear error interpretation is critical, given that DVH prediction error should inform the action thresholds for the clinician to accept or reject a candidate plan. In the case of at least one commercially available knowledge‐based planning system, RapidPlan™ (Varian Medical Systems, Palo Alto, CA), the lower DVH prediction error band limit not only guides clinician expectations for attainable dose sparing, but it also critically sets objectives for plan inverse optimization. If knowledge‐based DVH predictions are regarded as tests for plan optimality, then underestimated prediction error reduces test sensitivity (adequate plans deemed suboptimal), while overestimated prediction error reduces test specificity (suboptimal plans deemed adequate).

Fortunately, DVH prediction error is independently quantifiable and testable. In this work, we describe a general methodology to empirically determine DVH prediction error independently of the particular knowledge‐based model, using a reference cohort of high‐quality^3^ treatment plans. Then we apply this method to compare the prediction success rate of our empirically derived error bands to the model‐provided OAR DVH error bands. We examine two knowledge‐based models: ORBIT‐RT,^11^ a free, web‐based DVH prediction platform (www.orbit‐rt.com), and commercially available RapidPlan.

## 2 MATERIALS AND METHODS

A set of known high‐quality^3^ 135 volumetric modulated arc therapy (VMAT) prostate treatment plans from our Institution was available as the reference planning cohort. Ninety of these plans (training set) were used to train both the ORBIT‐RT and RapidPlan models. Both models were then used to generate OAR DVH predictions for the remaining 45 treatment plans (validation set, N=45).

The 45 predicted DVHs $V_{pred,i}\left(D\right)$ of the validation set were then compared to their analogous, clinically accepted DVHs $V_{clin,i}\left(D\right)$ to define prediction errors $V_{clin,i}‐V_{pred,i}$. As the DVHs are necessarily OAR volume normalized, all DVH error metrics are consequently also expressed as OAR volume percentages and as functions of dose. The dosewise mean error $\mu \equiv \bar{V_{clin,i}‐V_{pred,i}}$ serves as a metric for prediction bias, while the standard deviation *σ* of $V_{clin,i}‐V_{pred,i}$ indicates prediction error variation. Summation in quadrature of bias *μ* and error uncertainty *σ* yield the root‐mean‐square error of the predictions, $RMSE_{\mathrm{pred}}$:

(1) $$\begin{array}{c} RMSE_{\mathrm{pred}}\left(D\right)\equiv \sqrt{\frac{1}{N}\sum_{i}{\left(V_{clin,i}\left(D\right)‐V_{pred,i}\left(D\right)\right)}^{2}}≅\sqrt{\sigma^{2}+\mu^{2}}\\ where\\ \sigma \equiv \sqrt{\frac{1}{N‐1}\sum_{i}{\left(\left(V_{clin,i}\left(D\right)‐V_{pred,i}\left(D\right)\right)‐\mu \right)}^{2}}\;and\;\mu \equiv \bar{V_{clin,i}\left(D\right)‐V_{pred,i}\left(D\right)} \end{array}$$

In other words, the ORBIT‐RT and RapidPlan models’ accuracy were empirically sampled from the same set of independent DVH prediction “trials,” and the predictions’ resulting difference from the reference clinical values, quantified aggregately by $RMSE_{\mathrm{pred}}$. The values *μ*, *σ*, and $RMSE_{\mathrm{pred}}\left(\mu ,\sigma \right)$ serve as independent metrics for prediction accuracy because they are irrespective of the particular prediction model used. Furthermore, when μ≈0 in Eq. (1), $RMSE_{\mathrm{pred}}$ is readily interpretable as a canonical standard deviation, wherein we would expect to find 68% of an ideal, normal distribution of the reference cohort's 45 clinical DVHs, by the central limit theorem. $RMSE_{\mathrm{pred}}$ is a statistical outcome of the entire reference cohort, and is thus not patient specific.

**FIG. 1:**
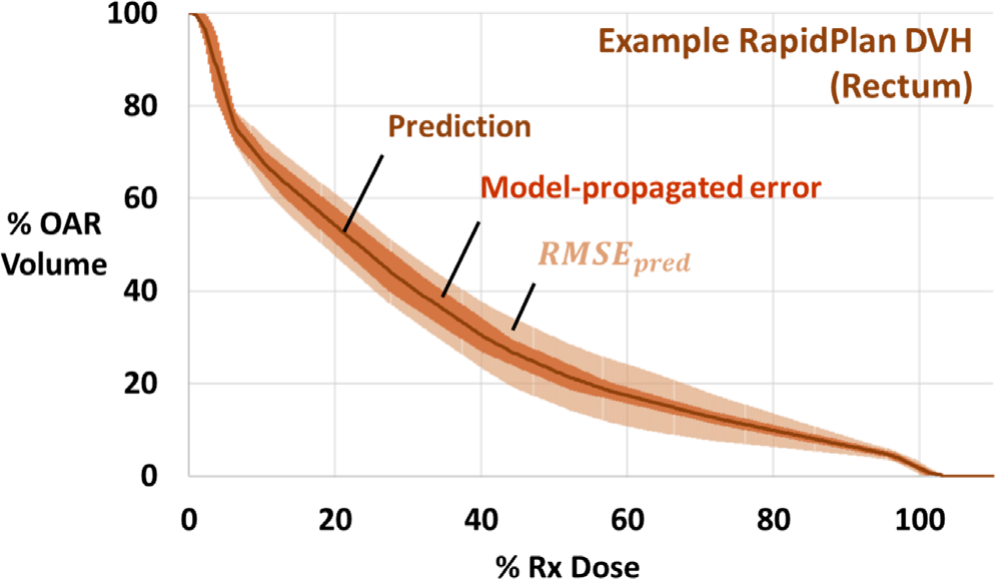
An example prostate OAR DVH prediction from RapidPlan, showing how different error estimation methods can alter clinician expectations for attainable dose sparing. The empirically derived $RMSE_{\mathrm{pred}}$ is universally calculable for any model and readily interpretable as a standard deviation when prediction bias is low [Eq. (1)], but it requires a validation set and may be overestimated for individual patients. The RapidPlan model‐propagated error does is tailored to the individual patient anatomy, but it may not always resemble a true standard deviation.

Both ORBIT‐RT and RapidPlan also provide their own patient‐specific DVH error estimates (Fig. 1), propagated from their trained models, which may better account for individual patient anatomy than the aggregate $RMSE_{\mathrm{pred}}$. In the case of ORBIT‐RT, the DVH prediction model is trained on plans based on their spectrum of target‐to‐organ distances. To obtain an error band, ORBIT‐RT considers separately the lower and upper sets of observed distances — [min, median] and [median, max] — within the training set, calculating separate DVHs to act as lower and upper error bounds, respectively. This error estimation formalism suggests a resemblance to an interquartile range (IQR), although it does not strictly meet that definition. Thus, the claim that 50% of clinical DVHs will be found within ORBIT‐RT prediction error becomes a testable hypothesis.

In the case of RapidPlan, DVH prediction error is propagated in quadrature from the standard error related to the regression model, weighted by the DVH principal components.^12^ This error is added and subtracted from the predicted DVH to create a symmetric error band. The RapidPlan error estimate is described in Ref. [12] as a standard deviation; as such, we expect a prediction success rate very similar to $RMSE_{\mathrm{pred}}$, or approximately 68% of clinical DVHs within prediction error.

$RMSE_{\mathrm{pred}}$ thus serves two purposes in this analysis: (a) as an independent metric for quantifying any model's DVH prediction accuracy and uncertainty, and now additionally (b) as a familiar reference value (*σ*) from which to relate the sensitivity and specificity of the models’ own provided DVH error bands. The 45 clinical DVHs of the reference cohort were tallied, to quantify how many cases successfully fell within the two types of DVH prediction error bands for each model: the empirical $RMSE_{\mathrm{pred}}$ bands, and the model‐propagated error bands provided. Clinical DVHs were tallied as correctly predicted if they were either within the prediction error band or less than 0.5% of OAR volume from the prediction (i.e., within clinically relevant dose‐volume granularity).

## 3 RESULTS

**FIG. 2:**
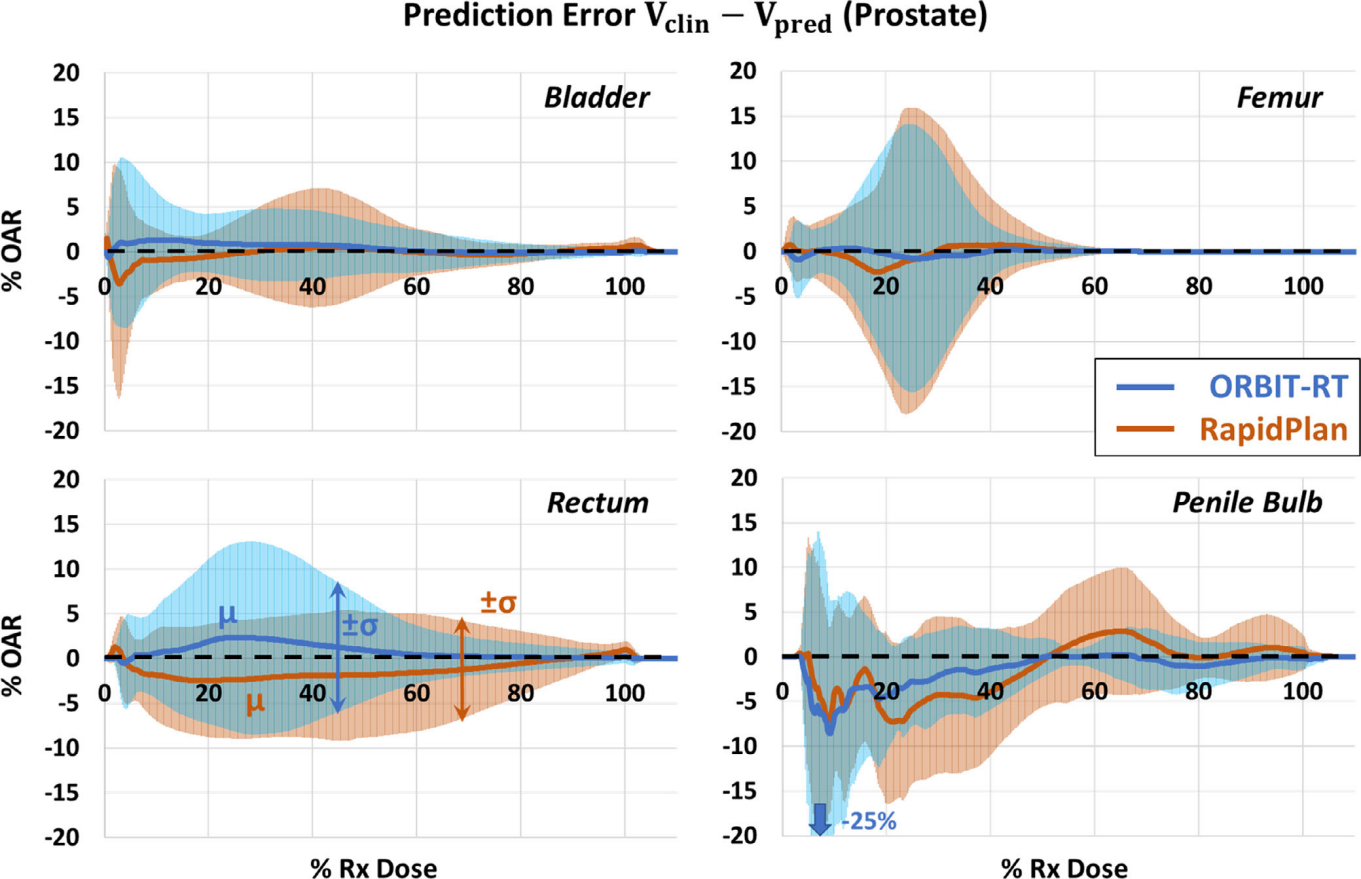
ORBIT‐RT and RapidPlan prediction bias and variation are directly compared for a validation set of 45 prostate treatment plans and 5 OARs. Mean errors (bias, μ) are represented as curves, and error standard deviations (variation, σ) are represented as symmetric bands about the curves. ORBIT‐RT prediction bias and variation (blue) are less than or comparable to those of RapidPlan (orange) for all examined OARs above 50% Rx dose.

**FIG. 3:**
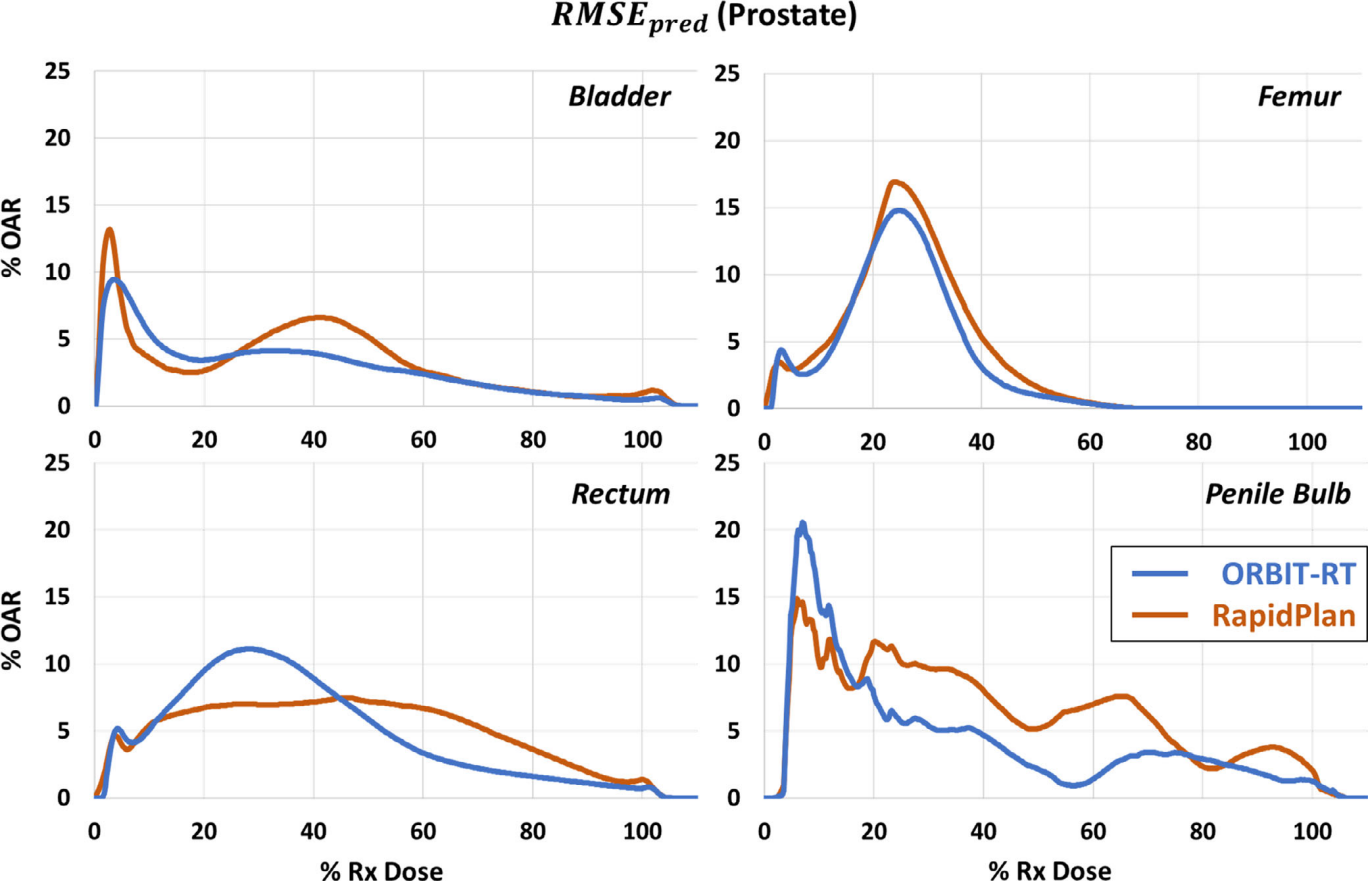
Lower prediction bias and variation yield lower $RMSE_{\mathrm{pred}}$ and greater prediction accuracy [Eq. (1)]. For a prostate validation set of 45 treatment plans, ORBIT‐RT $RMSE_{\mathrm{pred}}$ (blue) is less than or comparable to RapidPlan $RMSE_{\mathrm{pred}}$ (orange) above 50% Rx dose, indicating slightly improved prediction accuracy.

**FIG. 4:**
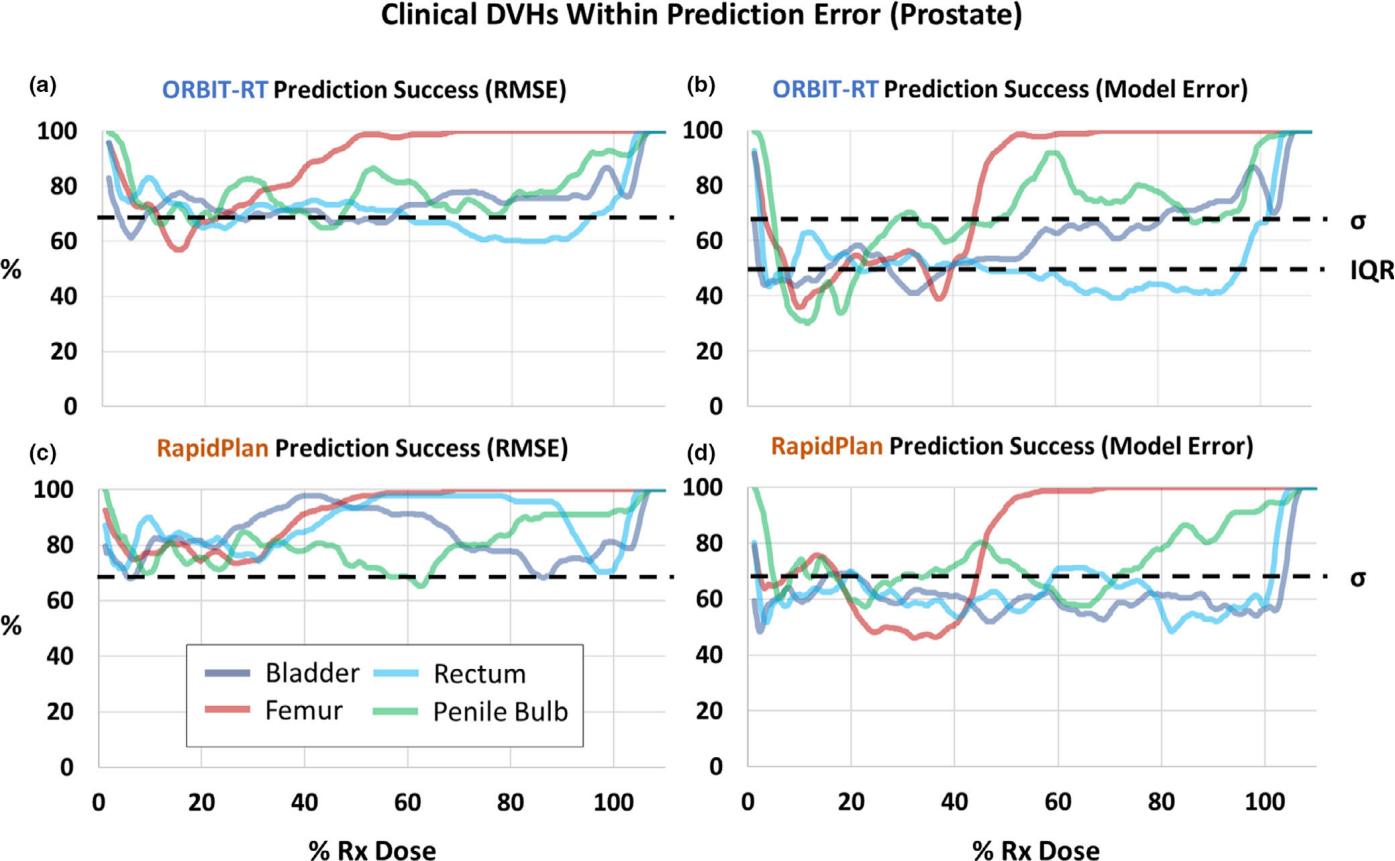
Clinical prostate OAR DVHs successfully predicted by their analogous predictions’ error bands were tallied as a percentage of the total validation set. An 11‐point boxcar smoothing routine has been applied to the data. As expected, by Eq. (1), both ORBIT‐RT and RapidPlan empirical $RMSE_{\mathrm{pred}}$ bands (a, c) successfully predict clinical DVHs at a frequency typical of σ or greater. Meanwhile, the ORBIT‐RT model‐propagated error band more closely resembles an IQR (b), and the RapidPlan model‐propagated error band is slightly less predictive than σ (d).

Following the prescribed methodology, we first examine the models’ prediction bias *μ* and error uncertainty *σ* (Fig. 2). Then we see how *μ* and *σ* contribute to $RMSE_{\mathrm{pred}}$ (Fig. 3), our independent metric for model accuracy. Finally, we compare the provided error bands of ORBIT‐RT and RapidPlan to our $RMSE_{\mathrm{pred}}$, by quantifying their prediction success rates (Fig. 4).

Figure 2 compares *μ* and *σ* of ORBIT‐RT and RapidPlan DVH predictions for five OARs of the prostate validation set. Above 40% Rx dose, ORBIT‐RT $\left|\mu \right|<1\%$ of OAR volume for all OARs, indicating little to no trend toward overprediction (*μ* < 0) or underprediction (*μ* > 0). In the same dose interval, RapidPlan predictions exhibited slightly more bias for certain OARs, tending toward overprediction for the rectum (*μ* = −2%) and penile bulb (*μ* = −3%). In both models, bias all but vanished at 80% Rx dose and higher. Below 40% Rx dose, both models exhibited more persistent, albeit slight, bias in their rectum and penile bulb predictions.

There is greater distinction in *σ* between the ORBIT‐RT and RapidPlan models in Fig. 2. Above 50% Rx dose, *σ* for ORBIT‐RT was comparable to or less than that of RapidPlan. This was most clinically significant at 100% Rx dose, where RapidPlan predictions for the validation set varied as much as 1–2% of OAR volume for the bladder, rectum, and penile bulb. One exception to this trend was the low‐dose rectum, known to have large error,^8^ in which *σ* for ORBIT‐RT was greater than RapidPlan below 50% Rx dose.

Using Eq. 1 and our subsequent observations of *μ* and *σ*, we now examine the empirical prediction error estimate $RMSE_{\mathrm{pred}}$ for both models. Figure 3 summarizes $RMSE_{\mathrm{pred}}$ calculations for all OARs over the examined dose interval. Above 50% Rx dose, in the clinically relevant prostate dose interval, ORBIT‐RT predictions exhibited comparable or slightly lower $RMSE_{\mathrm{pred}}$ than RapidPlan, indicating slightly improved accuracy. Below 50% Rx dose, the relative accuracy between the two models was more variable.

When *μ* ≈ 0, as verified in Fig. 2 for much of the dose interval, $RMSE_{\mathrm{pred}}\approx \sigma$. This makes $RMSE_{\mathrm{pred}}$ readily interpretable as a canonical standard deviation of prediction error, and we should expect about 68% of normally distributed clinical DVHS within the error band, $V_{\mathrm{pred}}\pm RMSE_{\mathrm{pred}}$. This was confirmed in Figs. 4(a) and 4(c) for both ORBIT‐RT and RapidPlan DVH predictions, where the $RMSE_{\mathrm{pred}}$ band was reliably predictive 68% of the time or greater. By contrast, both ORBIT‐RT and RapidPlan model‐propagated DVH error bands [Figs. 4(b) and 4(d)] were observed to be less predictive than $RMSE_{\mathrm{pred}}\approx \sigma$. Across the entire dose interval, ORBIT‐RT's prediction success was in the range of 40‐65%, while RapidPlan's prediction success was in the range of 55–70%.

## 4 DISCUSSION

$RMSE_{\mathrm{pred}}$ was proposed as a model‐independent metric for quantifying DVH prediction accuracy and uncertainty. By this metric, Figs. 2 and 3 demonstrated that ORBIT‐RT and RapidPlan were quite comparable in their prostate OAR prediction accuracy in the clinically relevant > 50% Rx dose range, with ORBIT‐RT at times more accurate, such as with the rectum and penile bulb. Below 50% Rx dose, increased variation in the prostate training set likely resulted in more varied (but still quite comparable) relative accuracy between the models, since prostate plan quality evaluations emphasize the >50% Rx dose interval. The comparability of $RMSE_{\mathrm{pred}}$ validates the use of ORBIT‐RT as equivalent to RapidPlan for prostate OAR DVH predictions at our Institution. Other institutions may similarly assess the accuracy of these models using their own reference cohorts, where interinstitutional variations in treatment planning may yield different results for $RMSE_{\mathrm{pred}}$.

The model‐propagated error bands of both models were found to capture clinical DVHs less frequently than our empirical error band $V_{\mathrm{pred}}\pm RMSE_{\mathrm{pred}}$ (Fig. 4), which was shown to perform in line with a canonical standard deviation. For ORBIT‐RT, this was hypothesized; at 40‐65% predictive success, ORBIT‐RT's error band more closely resembles an IQR, as described in Ref. [11]. The predictive success of RapidPlan's error band, described in Ref. [12] as a standard deviation, should have been similar to $RMSE_{\mathrm{pred}}$, but instead ranged lower, from 55 to 70%.

An ample retrospective validation set was required to calculate $RMSE_{\mathrm{pred}}$, enabling confident quantification of ORBIT‐RT's and RapidPlan's prostate OAR DVH prediction accuracy. Moreover, the validation exercise must be repeated for any new disease site considered. This exercise and analysis are standard protocol for every disease site model prior to its availability on ORBIT‐RT. Clinicians who wish to assess OAR DVH prediction accuracy themselves must have access to a broadly sampled set of past treatment plans to efficiently estimate $RMSE_{\mathrm{pred}}$.

## 5 CONCLUSIONS

A methodology has been established for comparing the accuracy of different DVH prediction models in a standardized way, using $RMSE_{\mathrm{pred}}$ as a familiar statistical metric. On this basis, ORBIT‐RT OAR DVH predictions were closely comparable to those of RapidPlan for a prostate validation set, validating its use as a free alternative to RapidPlan.

ORBIT‐RT and RapidPlan calculate their own patient‐specific prediction error, provided to the clinician while planning. These model‐propagated error estimates are often less than the empirical $RMSE_{\mathrm{pred}}$ of our analysis, suggesting that both model‐propagated error bands are generally less predictive than a canonical standard deviation *σ*. As hypothesized, ORBIT‐RT's error band is best described as an IQR. As such, clinicians relying on ORBIT‐RT and RapidPlan DVH prediction error estimates may need to exercise judgment when deciding whether further OAR sparing as suggested by the model is likely attainable. Independent quantification of prediction error with a validation set, as prescribed in this work with $RMSE_{\mathrm{pred}}$, may be valuable when enough retrospective plans are locally available.

## AUTHOR CONTRIBUTIONS

B. M. Covele is the primary author of this work. He is responsible for the development of the ORBIT‐RT source code, including DVH predictions and error estimates. He also wrote the analysis script for calculating the RMSE of each knowledge‐based model's DVH predictions, quantifying the prediction success rate, and plotting and interpreting the results.

C. J. Carroll provided guidance on the methodology in which ORBIT‐RT may generate its own model‐propagated error estimate, which has now been made a permanent module of the ORBIT‐RT source code. He also investigated a variant of the ORBIT‐RT DVH prediction model using Wasserstein‐Frechet means in log‐quantile density space, although this variant yielded no significantly improved prediction accuracy over the standard arithmetic mean.

K. L. Moore is the Principal Investigator for this work. He is responsible for building both the training and validation sets of prostate treatment plans, enabling the analysis of DVH prediction accuracy. He also provided guidance on those plots which would be most illustrative of DVH prediction accuracy, defining a template for the reported results.
